# Letter to the editor: Viral load decline time offers theoretical insight rather than practical value

**DOI:** 10.2807/1560-7917.ES.2025.30.9.2500151

**Published:** 2025-03-06

**Authors:** Nawfal R. Hussein, Delovan S. Mahfodh

**Affiliations:** 1 Department of Biomedical Sciences, College of Medicine, University of Zakho, Duhok, Iraq; 2 Department of Medicine, College of Medicine, University of Duhok, Duhok, Iraq

**Keywords:** Viral Load, Infectiousness, Transmission Risk

**To the editor**: We read with great interest the study by Jonnerby et al. [1] that investigated the correlation between viral load (VL) decline time and transmission risk for severe acute respiratory syndrome coronavirus 2 (SARS-CoV-2) and influenza A virus. We argue that the conclusions are scientifically uncertain and impractical for real-world application and that the study findings provide theoretical insight rather than practical value due to several reasons that follow.

First, host genetic variability is a major confounder in assessing VL decline time as a predictor of infectiousness, as it considerably influences immune responses and viral clearance rates. The research assumes that VL decline time uniformly predicts infectiousness. However, with regards to this, the consideration that human genetic diversity might substantially shape immune responses and affect viral clearance rates may have been important [2,3]. Though overlooked, inherent immunological variations (e.g. interferon response) and genetic differences (e.g. human leukocyte antigen alleles) may greatly influence VL dynamics [4,5]. Moreover, adaptive immunity derived from past infections or previous exposure to the viruses, people’s use of drugs such as immune suppressive medications, and metabolic effects may introduce additional variability in VL reduction [6,7]. Such factors should be considered, and they make VL decline time as an unreliable marker of transmission risk.

Secondly, viral genetic variability and mutation rates may play a critical role in influencing transmission dynamics and VL decline rates. Severe acute respiratory syndrome coronavirus 2 variants (pre-Alpha, Alpha, Delta, Omicron) are distinct in replication kinetics, immune evasion mechanisms, and infectivity periods [8]. Those characteristics play crucial roles affecting VL decline rates. Likewise, tissue tropism and shedding patterns of influenza A virus subtypes (H1N1, H3N2) are not the same [9]. Dealing with those variants and subtypes as homogeneous entities ignores their biological diversity and compromises the validity of the study’s findings.

Thirdly, a practical limitation makes the results unsuitable for public health. Supposing that VL decline time is a strong predictor, its real-world utility is minimal because the transmission occurs before VL decline can be assessed [10], making VL decline time a retrospective, rather than predictive, marker. Furthermore, mass testing declines after epidemic peaks making longitudinal VL measurement impractical.

Finally, careful examination of the study report yields findings of some wide 95% confidence intervals (e.g.  − 6.9% to 95% for SARS-CoV-2 secondary attack rate), suggesting uncertainty. Moreover, the project by Jonnerby et al. [1] should have addressed the small sample size of participants recruited in the study with the lack of confirmation of transmission chains for all participants, which may have led to potential misclassification of transmission events.

Overall, although the study enhances our knowledge of viral kinetics, it fails to have practical relevance. The time for VL decline is overly prolonged, highly inconsistent, and impractical as a reliable predictor of infectiousness. More studies are needed to focus on real-time, actionable indicators.
